# Network meta-analysis of high tibial osteotomy combined with different bone defect filler materials for medial compartment knee osteoarthritis

**DOI:** 10.1186/s13018-025-06306-w

**Published:** 2025-10-21

**Authors:** Zhiwei Pei, Aixian Tian, Baoxin Zhang, Zijian Lian, Xinlong Ma

**Affiliations:** 1 Tianjin Hospital, Tianjin University, Jiefang Nan Road 406, Hexi District, Tianjin, 300211 People’s Republic of China; 2 Orthopedic Research Institute, Tianjin Hospital, Tianjin University, Jiefang Nan Road 406, Hexi District, Tianjin, 300211 People’s Republic of China; 3 The Second Affiliated Hospital of Inner Mongolia Medical University, No. 59, Kerqin South Road, Saihan District, Hohhot, 010010 Inner Mongolia People’s Republic of China

**Keywords:** High tibial osteotomy, Network meta-analysis, Knee osteoarthritis, Bone defect filler materials

## Abstract

**Objective:**

This study aims to systematically review and analyze the clinical and radiographic outcomes of high tibial osteotomy (HTO) combined with various bone defect filler materials for the treatment of medial compartment knee osteoarthritis (MCKOA). In light of the increasing adoption of enhanced recovery after surgery (ERAS) principles, we further seek to refine treatment strategies for MCKOA by comparing the therapeutic effects of different filler materials.

**Methods:**

We performed a comprehensive literature search of studies published from database inception to July 1, 2024, across PubMed, Medline, Embase, the Cochrane Library, Web of Science, and Scopus. Using a network meta-analysis framework, we systematically assessed clinical comparative studies involving two or more bone defect filler materials in patients undergoing medial open-wedge high tibial osteotomy. Evidence was synthesized through a Bayesian random-effects network meta-analysis. Methodological quality and risk of bias of included studies were evaluated using the Cochrane Risk of Bias Tool 2.0 and a modified Newcastle–Ottawa scale.

**Results:**

After rigorous screening, 29 eligible trials were identified from 2755 references, including 10 randomized controlled trials (RCTs) and 19 non-randomized comparative trials (NCTs), involving a total of 1549 participants. These trials evaluated six intervention types: autografts, allografts, synthetic grafts, composite grafts, xenografts, and no grafts. Analyses based on outcomes such as clinical bone healing time, percentage of complete bone union, Lysholm knee score, infection, and delayed union, autogenous iliac bone grafting showed potential as a favorable option. However, considering correction of osteotomy defects and donor site morbidity, autogenous iliac bone grafting should be avoided. In contrast, synthetic grafts or allografts appeared more favorable when assessed using outcomes like WOMAC score, lateral cortical fracture, and donor site morbidity.

**Conclusion:**

Taking into account postoperative prognosis and complication profiles in HTO patients, autogenous iliac bone grafting may demonstrates superior efficacy in treating MCKOA, though its associated risks warrant careful consideration. Synthetic grafts and allografts offer a more balanced risk–benefit profile and represent recommended alternatives.

**Level of evidence:**

Level III, a meta-analysis of Level I RCTs and Level II-III non-randomized controlled trials.

**Supplementary Information:**

The online version contains supplementary material available at 10.1186/s13018-025-06306-w.

## Introduction

Knee osteoarthritis (KOA), a prevalent and progressively severe joint disease among middle-aged and elderly populations, is frequently observed as medial compartment knee osteoarthritis (MCKOA) [1, 2]. The characteristic pathological features of MCKOA include significant wear of articular cartilage, bone hyperplasia, and narrowing of the joint space within the medial compartment of the knee, which significantly impair patients’ walking ability, daily activities, and overall quality of life [3, 4]. With the growing adoption of the enhanced recovery after surgery (ERAS) concept, increasing attention has been directed toward optimizing surgical outcomes, minimizing complications, and accelerating recovery, establishing it as a key research priority [5–7].

In recent years, medial opening-wedge high tibial osteotomy (MOWHTO) has demonstrated considerable success in treating MCKOA. By realigning the mechanical axis of the lower limb, MOWHTO effectively alleviates the load on the medial compartment, thereby improving joint function and patients’ quality of life [8, 9]. However, postoperative bone defects commonly encountered following MOWHTO represent a major challenge to surgical success. These defects may not only undermine the procedural effectiveness but also elevate the risk of nonunion, loss of correction, and ultimately impede rehabilitation progress [10–12]. Moreover, although initial results are often satisfactory, some patients may experience disease progression over time and eventually require total knee arthroplasty (TKA). A history of osteotomy can additionally increase the technical complexity of subsequent TKA [13, 14]. Therefore, precise preoperative planning, combined therapeutic strategies (e.g., addressing both bone defects and cartilage lesions), and optimization of surgical techniques are crucial for reducing complications and improving clinical outcomes [15, 16]. Evidence suggests that minor deviations in the osteotomy starting point (e.g., at 3 cm or 4 cm from the joint line) within an acceptable range do not significantly affect gap height or alignment correction. This flexibility helps reduce procedural variability, supports individualized application of the enhanced recovery after surgery (ERAS) protocol, and enhances operative adaptability [17].

To address the issue of bone defects following MOWHTO, researchers have extensively investigated various bone void fillers, including autografts, allografts, and synthetic materials. Each possesses distinct characteristics in promoting bone healing and maintaining mechanical stability, yet their effectiveness and optimal choice in MCKOA treatment remain controversial [18–20]. Autografts, particularly tricortical iliac crest grafts, are regarded as the gold standard due to their excellent osteogenic, osteoinductive, and osteoconductive properties [21–23]. However, their clinical application is constrained by donor site morbidity and restricted supply [24–26]. While allografts avoid donor site trauma, concerns remain about their mechanical stability, healing capacity, and the risk of disease transmission [27, 28]. Synthetic bone substitutes, such as calcium-based or phosphate-based materials, have demonstrated potential in clinical and radiological assessments, yet their efficacy continues to be debated [29–32]. Emerging options like composite fillers and xenografts (e.g., bovine xenografts) demonstrate potential but lack robust support from high-quality evidence [33–35].

Given that current research on MOWHTO combined with different bone void fillers for MCKOA mainly consists of single-center, small-sample randomized controlled trials, comparative studies evaluating the practical effects of various fillers remain insufficient [36–38]. Furthermore, traditional pairwise meta-analyses, which rely mainly on direct comparison models, provide preliminary conclusions on the efficacy of bone void fillers in MOWHTO but are limited in addressing the relative effectiveness among multiple interventions [39]. Therefore, this study employs Bayesian Random-Effects Network Meta-Analysis (NMA) to systematically evaluate the effectiveness of HTO combined with various bone void fillers in treating MCKOA. NMA allows for the simultaneous comparison of multiple interventions and synthesize both direct and indirect evidence within a complex comparative network, thereby offering a more comprehensive and accurate assessment of the relative effectiveness of different fillers in alleviating pain, improving joint function, and promoting bone healing [39].

This study aims not only to provide a scientific basis for clinicians to select appropriate fillers during MCKOA treatment, thereby optimizing surgical outcomes and patients’ quality of life. It also strives to ensure the accuracy and reliability of our findings through rigorous quality assessment of included studies. We anticipate that this study will contribute high-quality evidence to the field of MCKOA treatment, propel the development of relevant clinical practices, and provide robust support for the deeper integration of the ERAS concept in the management of knee osteoarthritis.

## Materials and methods

### Review protocol

This network meta-analysis (NMA) follows the guidelines provided in the PRISMA (Preferred Reporting Items for Systematic Reviews and Meta-Analyses) and AMSTAR (Assessing the methodological quality of systematic reviews) [40], aiming to comprehensively evaluate the efficacy of various bone defect filler materials in patients with medial compartment knee osteoarthritis undergoing medial opening wedge high tibial osteotomy (MOWHTO).

### Information sources and search strategy

We performed a systematic search was conducted across online databases including Medline, Embase, Cochrane Library, Web of Science, and Scopus up to July 5, 2024, to identify all eligible English-language studies. The search strategy centered around keywords such as “medial opening wedge high tibial osteotomy,” “bone graft,” “bone void filler,” “filling material,” and their synonyms. Additionally, the reference lists of all relevant articles were manually inspected to supplement the search results. The literature search was independently performed by two investigators, and any discrepancies were resolved through consultation with a review panel.

### Study selection

#### Studies were included if they met the following criteria

1. Clinical trials comparing any two or more distinct types of bone void fillers in patients undergoing isolated MOWHTO.
2. Trials providing detailed information on osteotomy gap size, fixation type (locked vs. non-locked plates), filler material type (nature and/or source), and deformity correction amount (on the mechanical axis).
3. Trials reporting at least one clinical or radiological outcome measure, including validated patient-reported outcomes (e.g., Knee Society Score), bone healing grades, bone/graft healing percentages, clinical time to bone union, and loss of correction angle at final follow-up.
4. Trials with a minimum follow-up duration of 12 months.

#### Exclusion criteria encompassed

1. Trials involving additional surgical procedures (e.g., anterior cruciate ligament reconstruction), revisions, or bilateral MOWHTO.
2. Grey literature, such as conference abstracts.

The study selection process involved an initial screening of titles and abstracts, followed by a full-text assessment of potentially eligible articles. Two researchers independently conducted the study selection, and disagreements were resolved by a third senior researcher.

### Data extraction and risk of bias assessment

#### Detailed data were extracted according to predefined criteria, encompassing

1. Trial characteristics, including study literature, location, study period, level of evidence, randomization and blinding, study duration, and follow-up losses.
2. Subject information, comprising patient numbers, age, gender, inclusion/exclusion criteria, and baseline correction angles (or gap widths).
3. Intervention details, such as osteotomy techniques, bone void filler types, fixation methods, postoperative rehabilitation, and weight-bearing protocols.
4. Relevant outcome measures.

For randomized controlled trials (RCTs), the Cochrane Risk of Bias Tool 2.0 was employed to assess bias risk, encompassing five domains: random sequence generation, allocation concealment, blinding of outcome assessment, completeness of outcome data, selective reporting, and other biases. For non-randomized controlled trials (NCTs), a modified Newcastle–Ottawa Scale was used to evaluate methodological quality (Supplementary Table 3). All assessments were independently conducted by three researchers, and disagreements were resolved through consensus and adjudication by the review panel.

### Outcome measures

The evaluated outcomes for various bone defect filler materials included time to bone union (TBU), percentage of complete bone union (PCBU), loss of correction (LOC), Lysholm knee score (LKS), Western Ontario and McMasters Universities Score (WOMAC), objective knee society scoring (OKSS), and functional knee society scoring (FKSS). Complication outcomes for different bone defect fillers included lateral cortical fracture (LCF), donor site morbidity (DSM), infection, and delayed union (DU).

### Transitivity and inconsistency assessment

Transitivity, a core assumption in NMA, requires interchangeability between studies comparing two treatments via a third. This was verified by meticulously comparing baseline characteristics across trials, including baseline correction angles, opening gap widths, sample sizes, age, body mass index (BMI), and the proportion of female patients. The similarity of these characteristics ensured the robustness of direct comparisons and provided a solid foundation for indirect comparisons.

Inconsistency detection was performed using the node-splitting method, with detailed results shown in Table 1 and Supplementary Table 1. This quantified local discrepancies between direct and indirect evidence for each treatment comparison. Bayesian *P*-values were calculated; results (*P* < 0.05) indicated inconsistency, necessitating supplementary random-effects adjustments. Model fit was validated using Deviance Information Criterion (DIC); lower DIC values denote preferable models (Table 1). This structured approach ensured robustness and transparency, providing a foundation for clinically interpretable indirect comparisons and treatment rankings.

**Table 1:** Comparisons of the fit of consistency and inconsistency models using DIC

Model	Outcome indicators										
	TBU	PCBU	LOC	LKS	WOMAC	OKSS	FKSS	LCF	DSM	Infection	DU
Consistency	31.9	37.8	51.7	32.4	31.3	34.2	37.9	28	2.4	15.7	20.6
Inconsistency	31.7	37.2	54.6	32.2	32.6	34.2	36.3	27.5	2.7	16.4	21.3

The DIC is a Bayesian model evaluation criterion that measures model fit adjusted with complexity of the model; smaller DIC values correspond to preferable models

DIC, deviance information criteria; TBU, time to bone union; PCBU, percentage of complete bone union; LOC, loss of correction; LKS, Lysholm knee score; WOMAC, Western Ontario and McMasters Universities score; OKSS, objective knee society scoring; FKSS; functional knee society scoring; LCF, lateral cortical fracture; DSM, donor site morbidity; DU, delayed union

### Treatment ranking metrics

To compare the relative efficacy of bone defect filler materials, we employed Bayesian treatment ranking based on posterior distributions from the network meta-analysis. Two primary metrics were used:

SUCRA (surface under the cumulative ranking curve), representing the probability of a treatment being among the most effective interventions (higher SUCRA values indicate superior efficacy). Mean Rank, reflecting the average rank position across posterior simulations. Rank probabilities were calculated for each outcome (e.g., WOMAC, bone healing rate) through 50,000 Markov Chain Monte Carlo (MCMC) iterations. Three key visualizations were programmatically generated:

Rankograms, illustrating probability distributions of all treatments occupying each rank position (1st to 6th); Rank Probability Plots, depicting per-treatment likelihoods of achieving specific ranks; Cumulative Ranking Plots, visualizing progressive superiority probability with uncertainty intervals. All computations and visualizations were implemented in R 4.4.0 using the gemtc package, ensuring reproducibility across outcomes.

### Data synthesis and statistical analysis

The Network Meta-Analysis (NMA) was conducted using the R package multinma [41], which incorporates Bayesian models and graph-theoretical approaches to synthesize both direct and indirect evidence. A network diagram was generated to illustrate the comparative relationships among the various treatment modalities included in the study. For continuous outcomes, such as knee society score (KSS), Western Ontario and McMaster Universities Osteoarthritis Index (WOMAC) score, correction loss, and clinical bone healing time, relative effects (REs) and their corresponding 95% credible intervals (CrIs) were employed as the metrics of evaluation. For binary outcomes, including complete or incomplete bone healing, odds ratios (ORs) and their 95% CrIs were utilized. Forest plots and rankograms of the relative treatment effects were utilized to visually represent the comparative results of the network estimates. A *P*-value of < 0.05 was considered statistically significant.

## Results

### Baseline characteristics of included studies

**Fig. 1:**
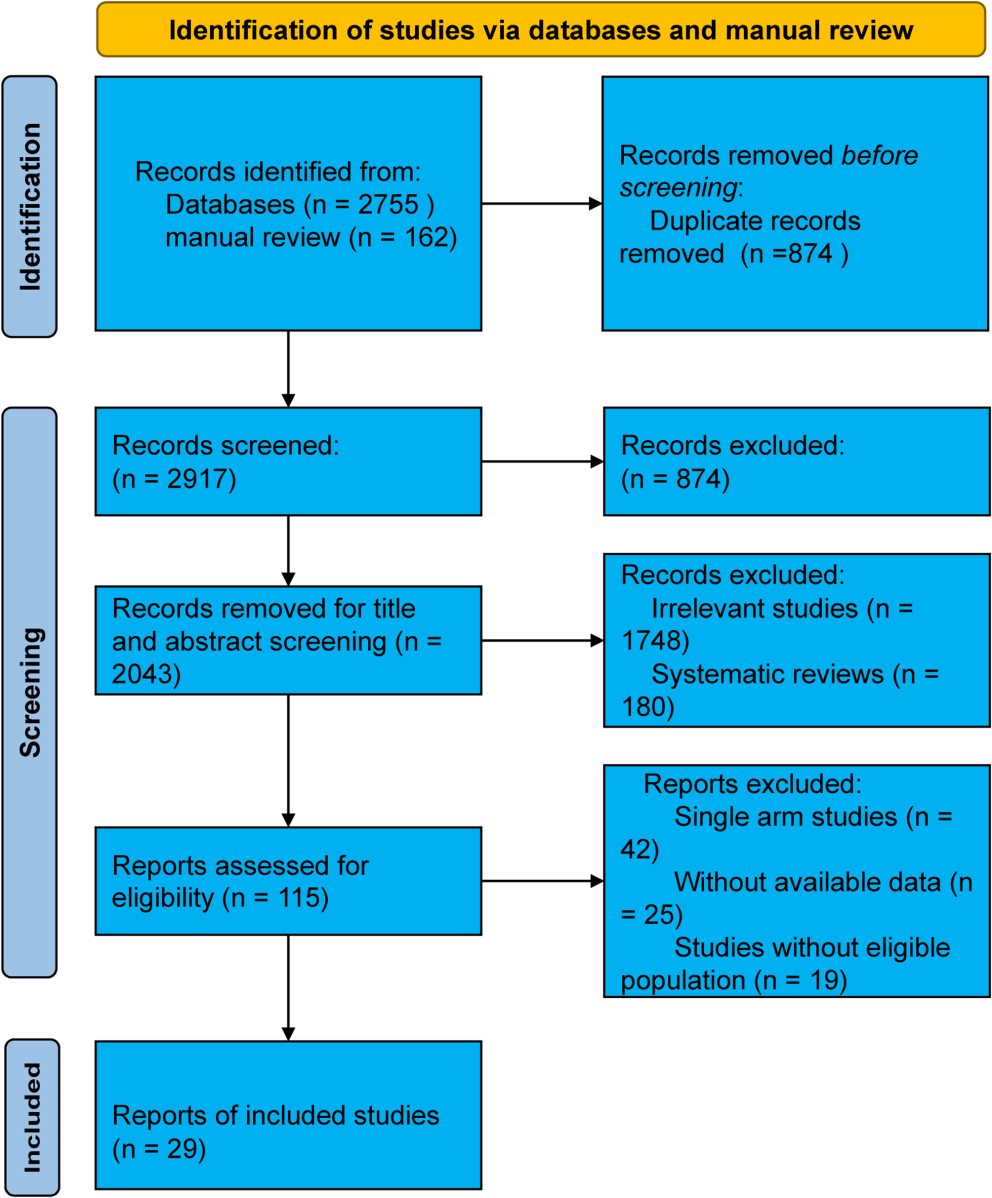
Flow diagram of study selection process

**Fig. 2:**
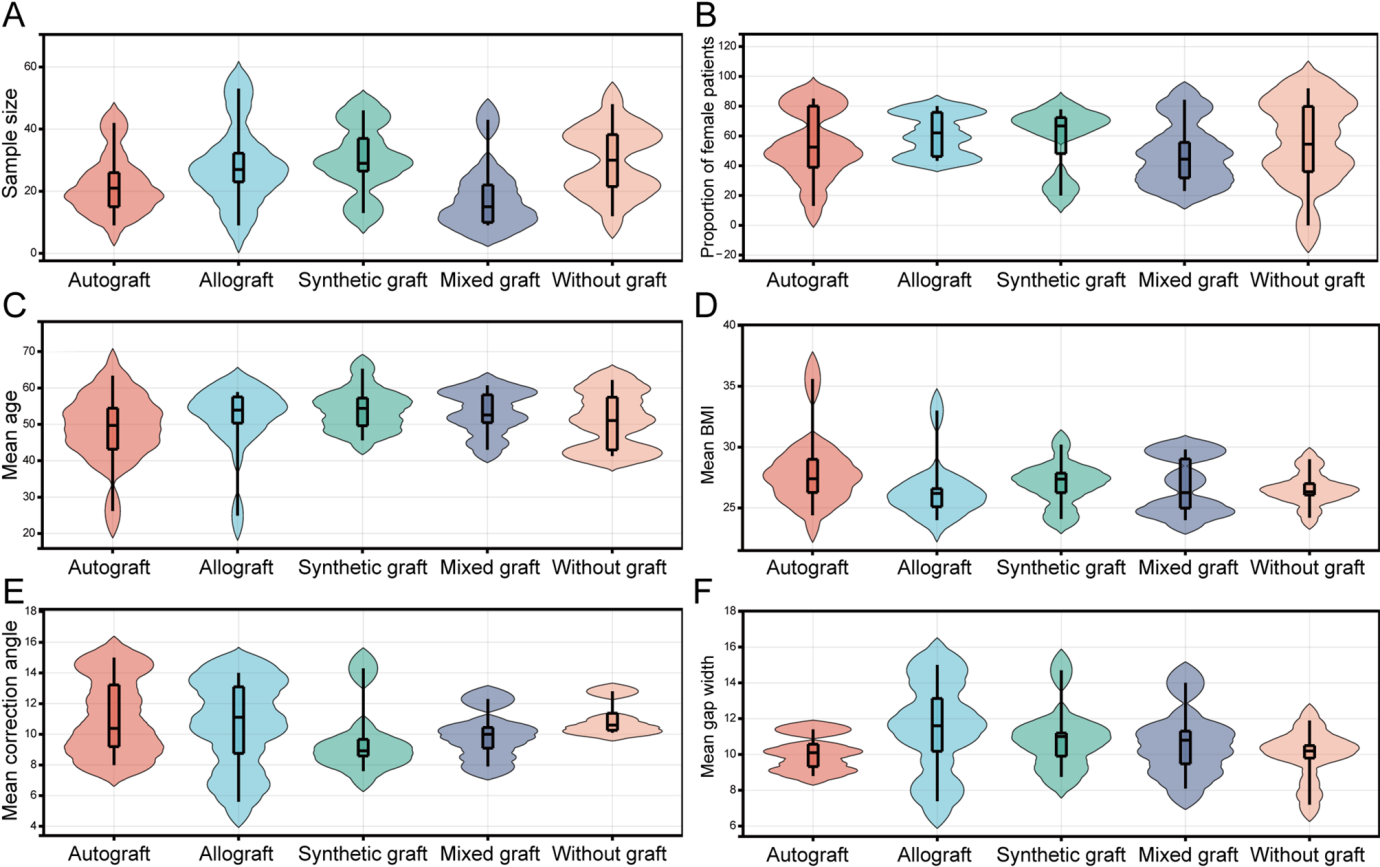
Violin plots of baseline characteristics distribution for different bone defect filler materials. **A** Sample size; **B** Proportion of female patients; **C** Mean age; **D** Mean BMI at baseline; **E** Mean correction angle at baseline; **F** Mean osteotomy gap width at baseline

A total of 2917 records were identified from the initial title and abstract screening, of which 115 full-text studies were retrieved and reviewed. Ultimately, 10 RCTs (n = 396) and 19 NCTs (n = 1153) met the inclusion criteria, comprising a total of 1549 patients and covering six distinct bone defect filler materials (Fig. 1). These included 334 patients with autografts, 375 with allografts, 354 with synthetic grafts (hydroxyapatite or calcium phosphate), 134 with hybrid grafts, 326 without bone grafts, and 26 with xenografts (Fig. 2). Among the participants, the mean age was 52.6 years (range: 24.9–65.3), with a female proportion of 54.55% (range: 0–91.89%), a mean baseline BMI of 26.6 (range: 24–35.6), and a median follow-up duration of 24 months (range: 12–96). The basic characteristics of all included studies are presented in Table 2 and Supplementary Table 2. There were some concerns regarding the overall risk of bias in the included RCTs, while the overall quality of the NCTs was relatively good (median score: 7). A detailed assessment of quality and risk of bias is summarized in Supplementary Table 3 and Supplementary Fig. 1.

**Table 2:** Summary of characteristics of included studies

Study characteristics	Median/No. (%)	IQR	Range
*Eligible studies*			
Total number of RCTs	10	–	–
Total number of NCTs	19	–	–
Number of participants	1549	–	–
Median follow-up, mo	24	12–28.43	12–96
*Region*			
Asia	17(58.62)	–	–
Europe	9(31.03)	–	–
North America	1(3.44)	–	–
South America	2(6.90)	–	–
Oceania	0	–	–
*Participants*			
Mean age, y	52.6	48.65–57.9	24.9–65.3
Percentage of females (%)	54.55	38.9–75	0–91.89
Baseline mean BMI	26.6	25.7–28.1	24–35.6
Intraoperative mean correction angle, °	10	8.6–12.46	5.6–15
Intraoperative mean gap width, mm	10.5	9.4–11.4	7.39–14.7

BMI, body mass index; IQR, interquartile range; RCT, randomized controlled trial; NCT, nonrandomized comparative trials

### Comparative outcomes of different bone graft materials in prognostic analysis

#### Prognostic analysis of clinical bone healing time (TBU) across bone graft materials

**Fig. 3:**
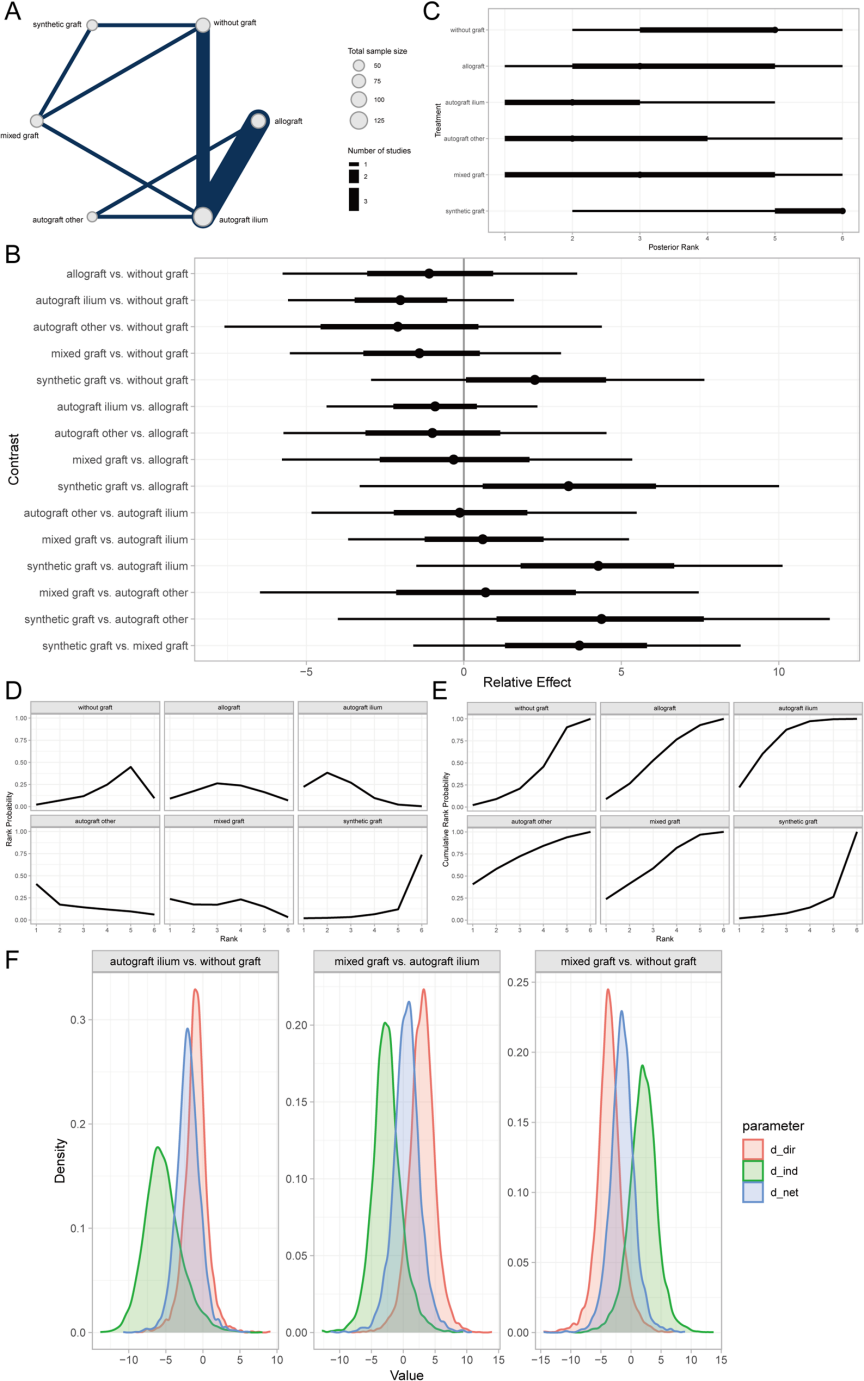
Prognostic analysis of clinical bone healing time across bone graft materials. **A** Network evidence diagram; **B** Forest plot of pairwise comparisons; **C** Intervention ranking plot; **D** Ranking probability plot for each intervention; **E** Cumulative ranking probability plot; **F** Local inconsistency analysis plot

Time to bone union (TBU) was defined as the absence of tenderness at the surgical site, painless full weight-bearing ambulation, or confirmation by radiological measurements [42]. Bayesian random-effects network meta-analysis revealed that autogenous iliac crest bone graft (AICBG) was associated with a shorter clinical bone healing time compared to the no-graft group, whereas synthetic grafts were associated with relatively increased healing times (Fig. 3A, B). Furthermore, the ranking and surface under the cumulative ranking curve (SUCRA) probabilities indicated that the prognosis for patients receiving AICBG was superior to other autogenous grafts (e.g., tibial cortical bone), hybrid grafts, allografts, no-graft, and synthetic grafts in descending order (Fig. 3C, E). Additionally, the local inconsistency analysis showed that the credible intervals (CrIs) of direct and indirect comparisons largely overlapped, with *P*-values exceeding 0.05, suggesting no significant local inconsistency (Fig. 3F).

#### Prognostic analysis of percentage of complete bone union (PCBU) at 1-year follow-up across bone graft materials

**Fig. 4:**
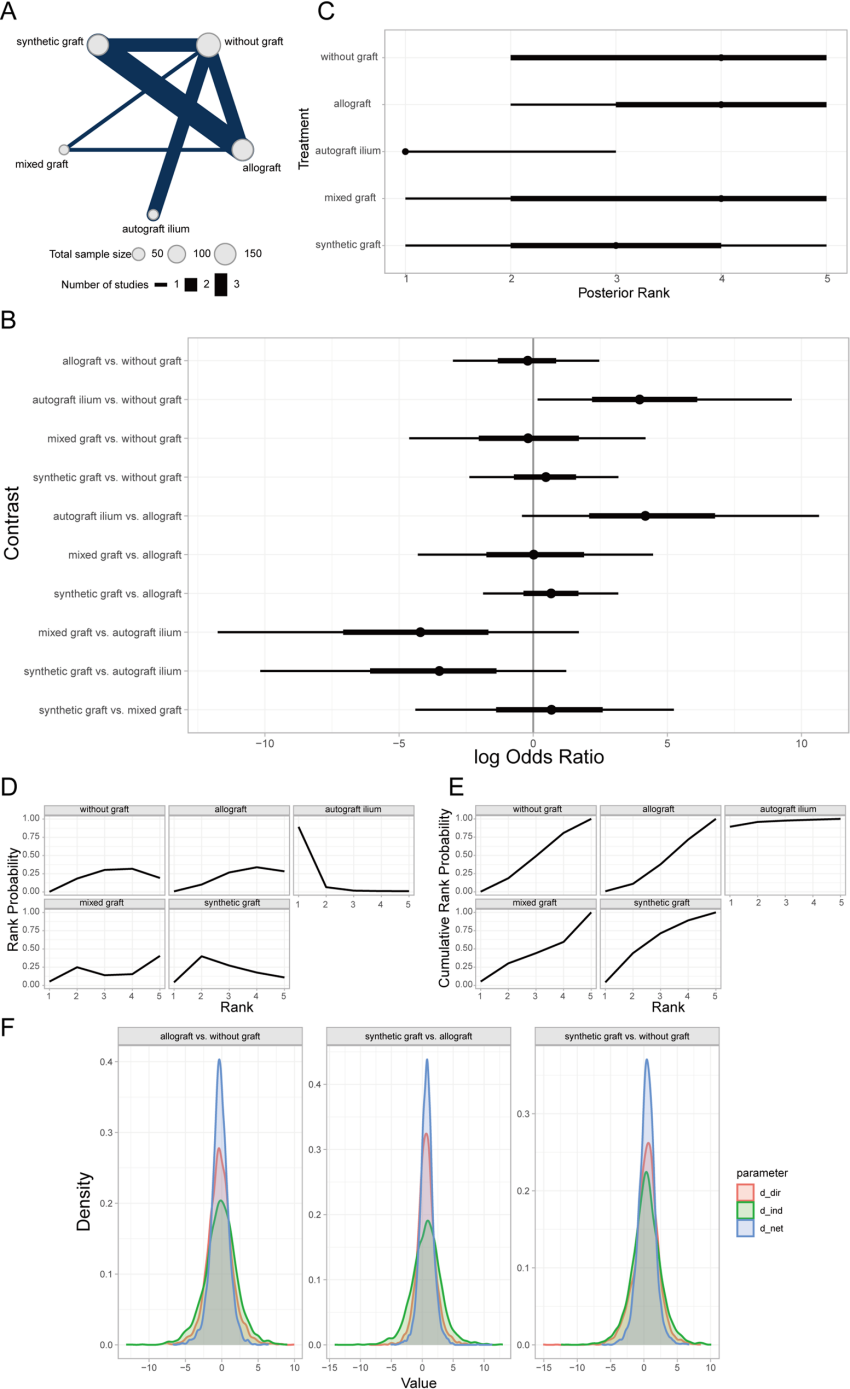
Prognostic analysis of PCBU at 1-year follow-up across bone graft materials. **A** Network evidence diagram; **B** Forest plot of pairwise comparisons; **C** Intervention ranking plot; **D** Ranking probability plot for each intervention; **E** Cumulative ranking probability plot; **F** Local inconsistency analysis plot

Complete bone union was defined as a van Hemert score of 5 (no radiolucent lines visible on any osteotomy surface) or a bone defect filling rate exceeding 95% on quantitative computed tomography (CT) at 1-year post-surgery. Otherwise, it was considered incomplete bone union [43]. The Bayesian random-effects network meta-analysis showed that AICBG was associated with a higher rate of complete bone union compared to the no-graft group (Fig. 4A, B). The ranking and SUCRA probabilities indicated that AICBG was superior to synthetic grafts, hybrid grafts, no-graft, and allografts in terms of prognosis (Fig. 4C–E). The local inconsistency analysis showed overlapping CrIs and *P*-values > 0.05 for direct and indirect comparisons, indicating no significant local inconsistency (Fig. 4F).

#### Prognostic analysis of loss of correction (LOC) across bone graft materials

**Fig. 5:**
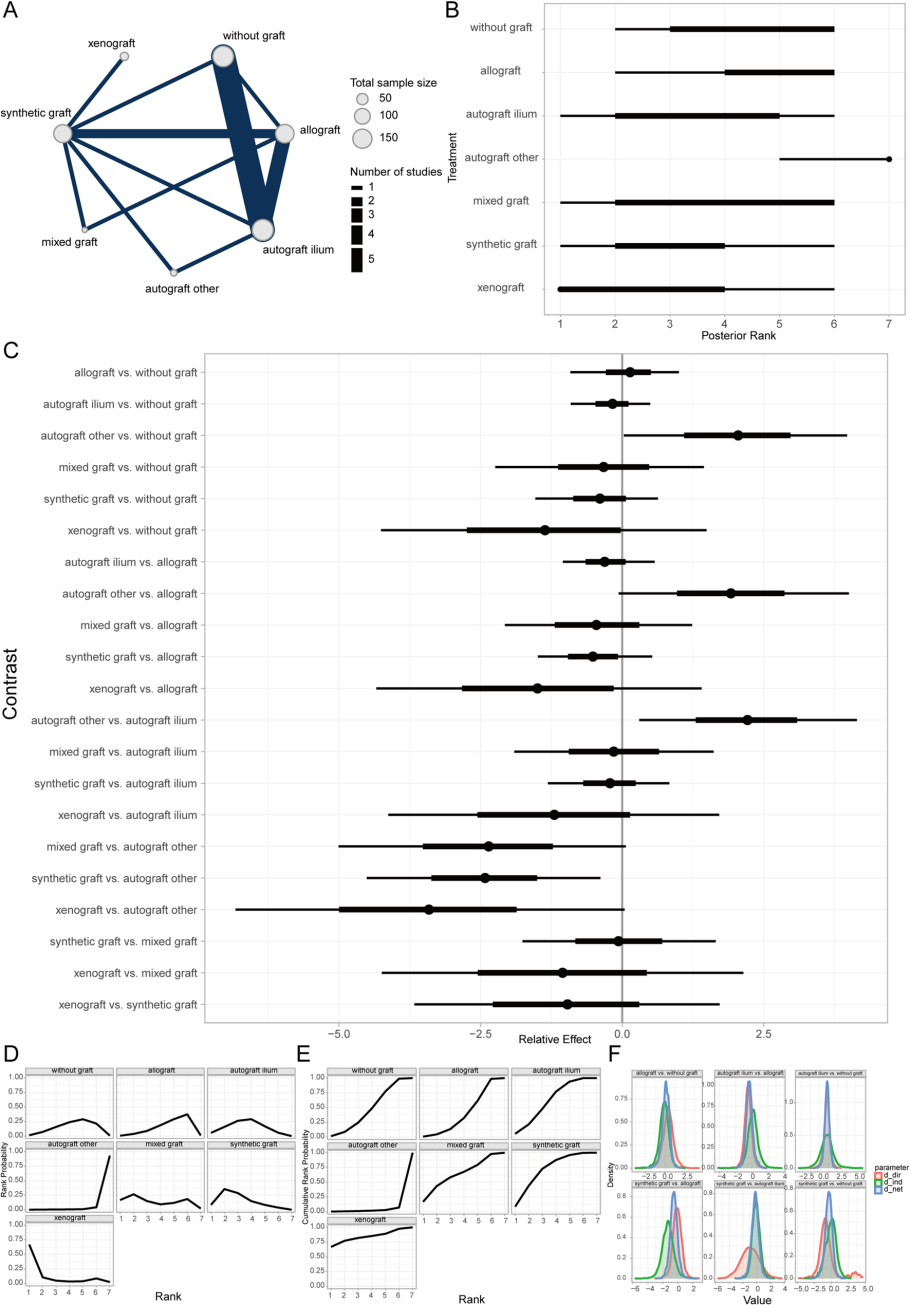
Prognostic analysis of LOC across bone graft materials. **A** Network evidence diagram; **B** Forest plot of pairwise comparisons; **C** Intervention ranking plot; **D** Ranking probability plot for each intervention; **E** Cumulative ranking probability plot; **F** Local inconsistency analysis plot

Loss of correction (LOC) was assessed by measuring the difference between the actual achieved correction angle and the preoperative planned or ideal correction angle at the final follow-up after HTO [44]. The Bayesian random-effects network meta-analysis indicated that AICBG showed a greater magnitude of LOC compared to the no-graft group (Fig. 5A, B). The ranking and SUCRA probabilities indicated that xenograft patients had the best prognosis, followed by synthetic grafts, hybrid grafts, AICBG, no-graft, allografts, and other autogenous grafts (e.g., tibial cortical bone) in descending order (Fig. 5C–E). The local inconsistency analysis found overlapping CrIs and *P*-values > 0.05, indicating no significant local inconsistency (Fig. 5F).

#### Prognostic analysis of different bone defect filler materials on Lysholm knee score

**Fig. 6:**
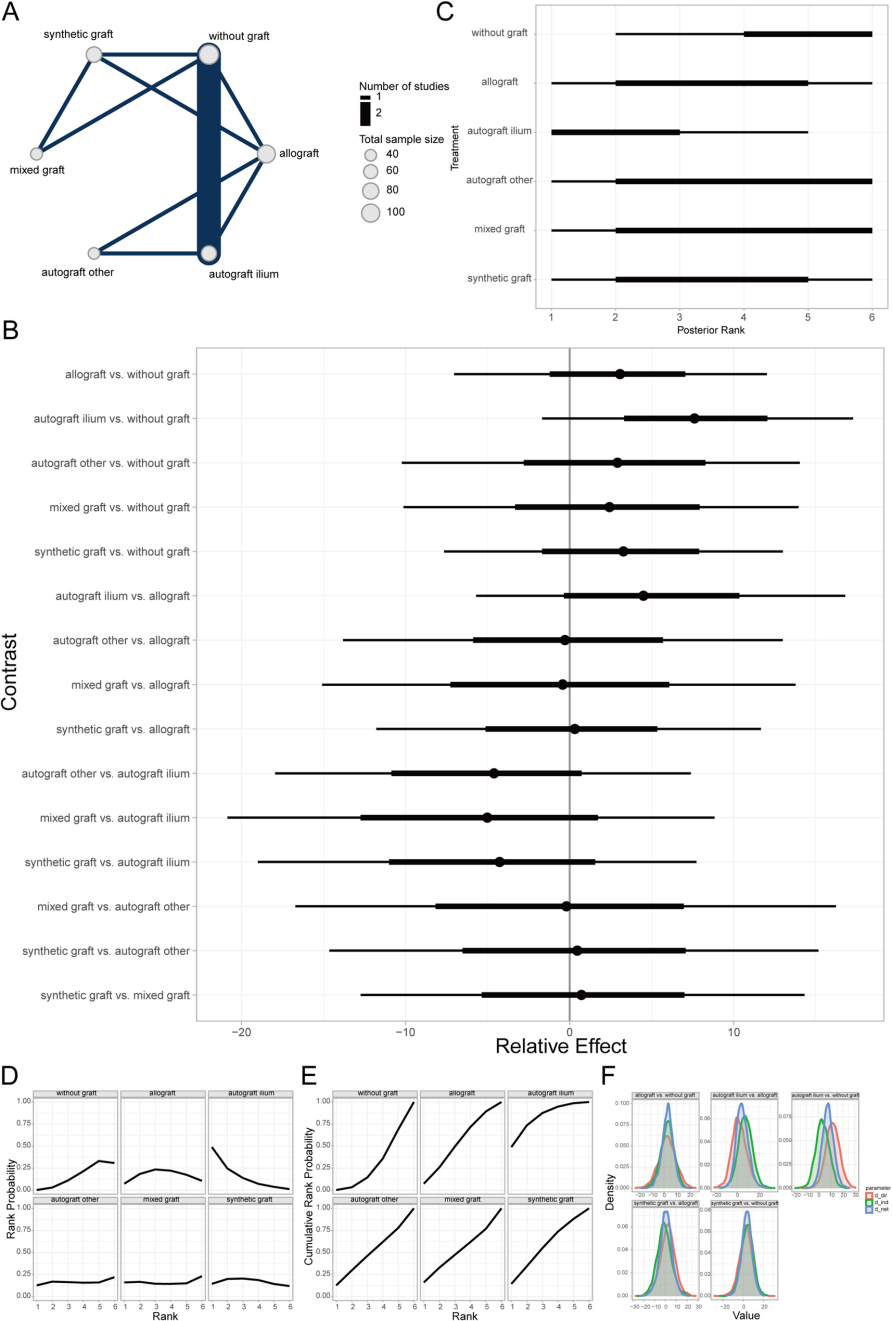
Prognostic analysis of different bone defect filler materials regarding lysholm knee score. **A** Network evidence diagram; **B** Forest plot of pairwise comparisons between interventions; **C** Intervention ranking plot; **D** Surface under the cumulative ranking curve (SUCRA) plot indicating the probability of each intervention being the best; **E** Cumulative ranking probabilities of interventions; **F** Local inconsistency analysis results

The Lysholm knee score serves as a quantitative assessment tool for evaluating the functional recovery of the knee joint, with a particular focus on pain and daily activity functions. Introduced by Lysholm and Gillqui in 1982, it has been extensively utilized in assessing the therapeutic outcomes of conditions such as knee ligament injuries, meniscal tears, cartilage degeneration, or softening [39]. Through a Bayesian random-effects network meta-analysis, we observed a relatively higher Lysholm knee score in patients who underwent autologous iliac bone grafting compared to those without any bone grafting (Fig. 6A, B).

Furthermore, intervention ranking and probability analysis indicated that autologous iliac bone grafting yielded sequentially better outcomes than synthetic material grafting, allografting, autologous grafting from other sites (e.g., tibial cortex), mixed grafting, and no grafting (Fig. 6C–E). Additionally, the local inconsistency analysis revealed that the Credible Intervals (CrIs) of direct and indirect comparisons largely overlapped, with *P*-values exceeding 0.05 for most comparisons (Fig. 6F). These findings suggest that there is no significant local inconsistency in the aforementioned results.

#### Prognostic analysis of different bone defect filling materials based on western ontario and mcmasters universities score (WOMAC)

**Fig. 7:**
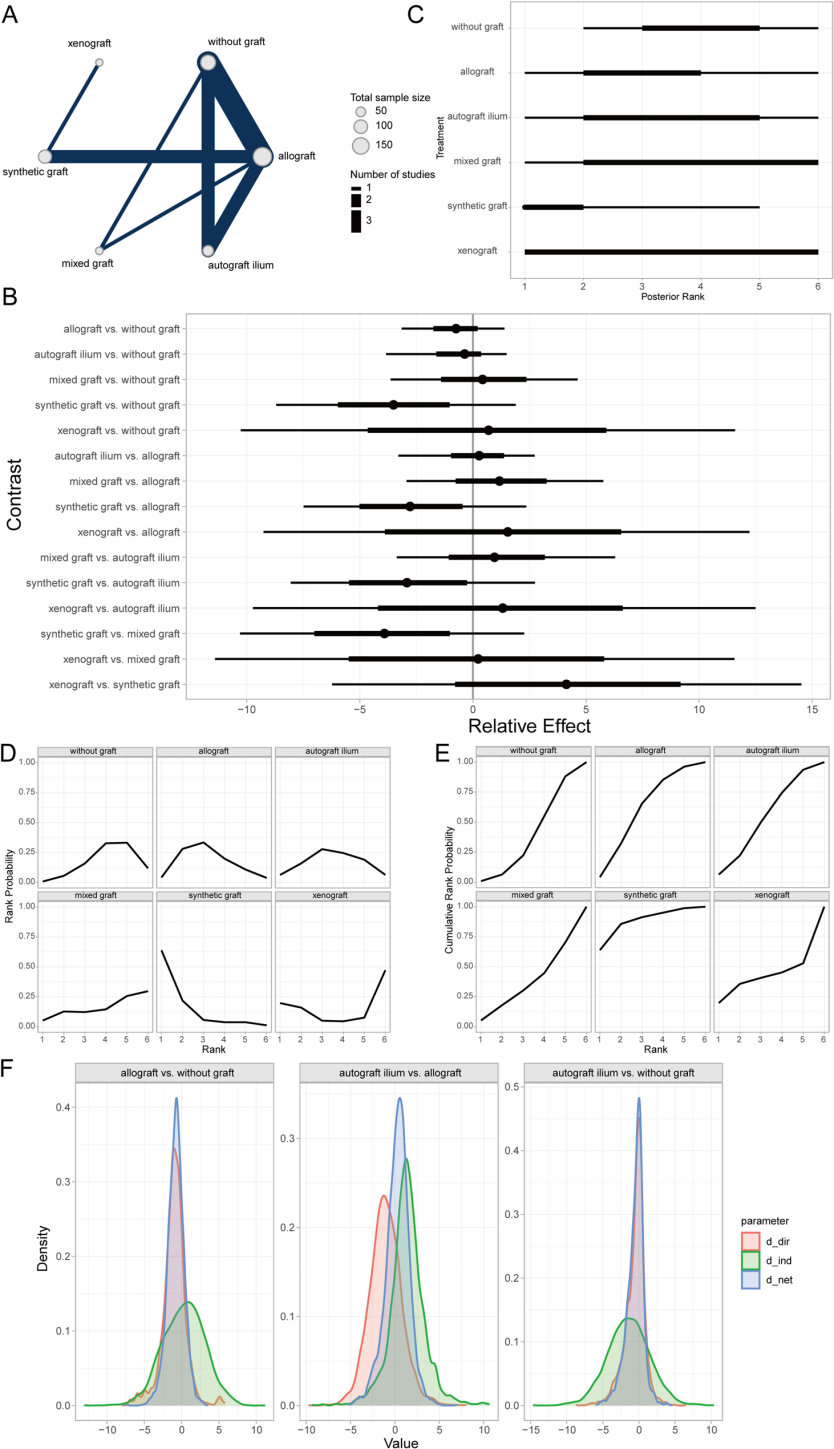
Prognostic analysis of different bone defect filling materials based on WOMAC Score. **A** Network evidence plot; **B** Forest plot of pairwise intervention comparisons; **C** Intervention ranking plot; **D** Probability of order plot for individual interventions; **E** Cumulative probability of order plot for individual interventions; **F** Local inconsistency analysis plot

The WOMAC score, a well-established clinical rating scale introduced by Bellamy and colleagues in 1988, is widely used to evaluate pain, stiffness, and joint function in patients with hip and knee osteoarthritis. Post-HTO (High Tibial Osteotomy), the WOMAC score is commonly adopted as a key metric for assessing surgical outcomes, with higher total scores indicating more severe knee pain, stiffness, and functional impairments [45]. Our Bayesian random-effects network meta-analysis revealed a relatively lower WOMAC score in patients who received synthetic material grafts compared to the non-graft group (Fig. 7A, B). Furthermore, the intervention rankings and probability of order analysis indicated that patients with synthetic material grafts had progressively better prognoses than those receiving xenogeneic, allograft, autogenous iliac bone grafts, non-graft, and composite grafts (Fig. 7C–E). Additionally, the local inconsistency analysis demonstrated substantial overlap in the CrIs of direct and indirect comparisons, with all *P*-values exceeding 0.05 (Fig. 7F), indicating no significant local inconsistency in the aforementioned findings.

#### Prognostic analysis of different bone defect filling materials based on objective knee society score (KSS)

**Fig. 8:**
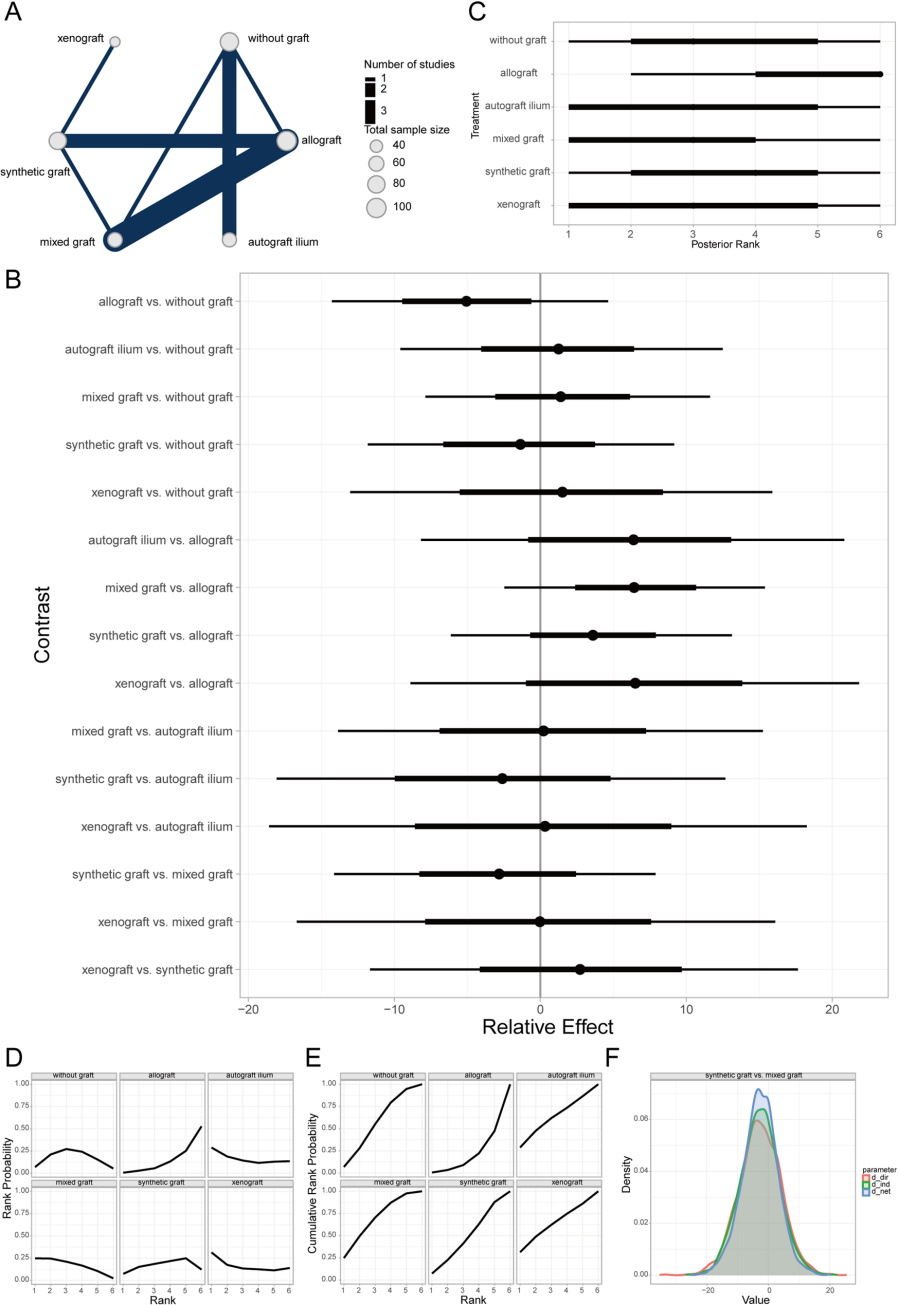
Prognostic analysis of different bone defect filling materials based on objective KSS. **A** Network evidence plot; **B** Forest plot of pairwise intervention comparisons; **C** Intervention ranking plot; **D** Probability of order plot for individual interventions; **E** Cumulative probability of order plot for individual interventions; **F** Local inconsistency analysis plot

The Knee Society Score (KSS), a validated patient-reported outcome measure, is used to evaluate outcomes after High Tibial Osteotomy (HTO). It comprises two parts: an objective score and a functional score, each ranging from 0 (worst) to 100 (best) [41]. Our Bayesian random-effects network meta-analysis indicated showed a lower Objective KSS score in patients receiving allografts compared to the non-graft group (Fig. 8A, B). The intervention rankings and probability of order analysis further revealed that patients with composite grafts had progressively better prognoses than those with xenogeneic, autogenous iliac bone grafts, non-graft, synthetic material grafts, and allografts (Fig. 8C–E). The local inconsistency analysis demonstrated substantial overlap in the CrIs of direct and indirect comparisons, with all *P*-values exceeding 0.05 (Fig. 8F), suggesting no significant local inconsistency in the results.

#### Prognostic analysis of different bone defect filling materials based on functional knee society score (KSS)

**Fig. 9:**
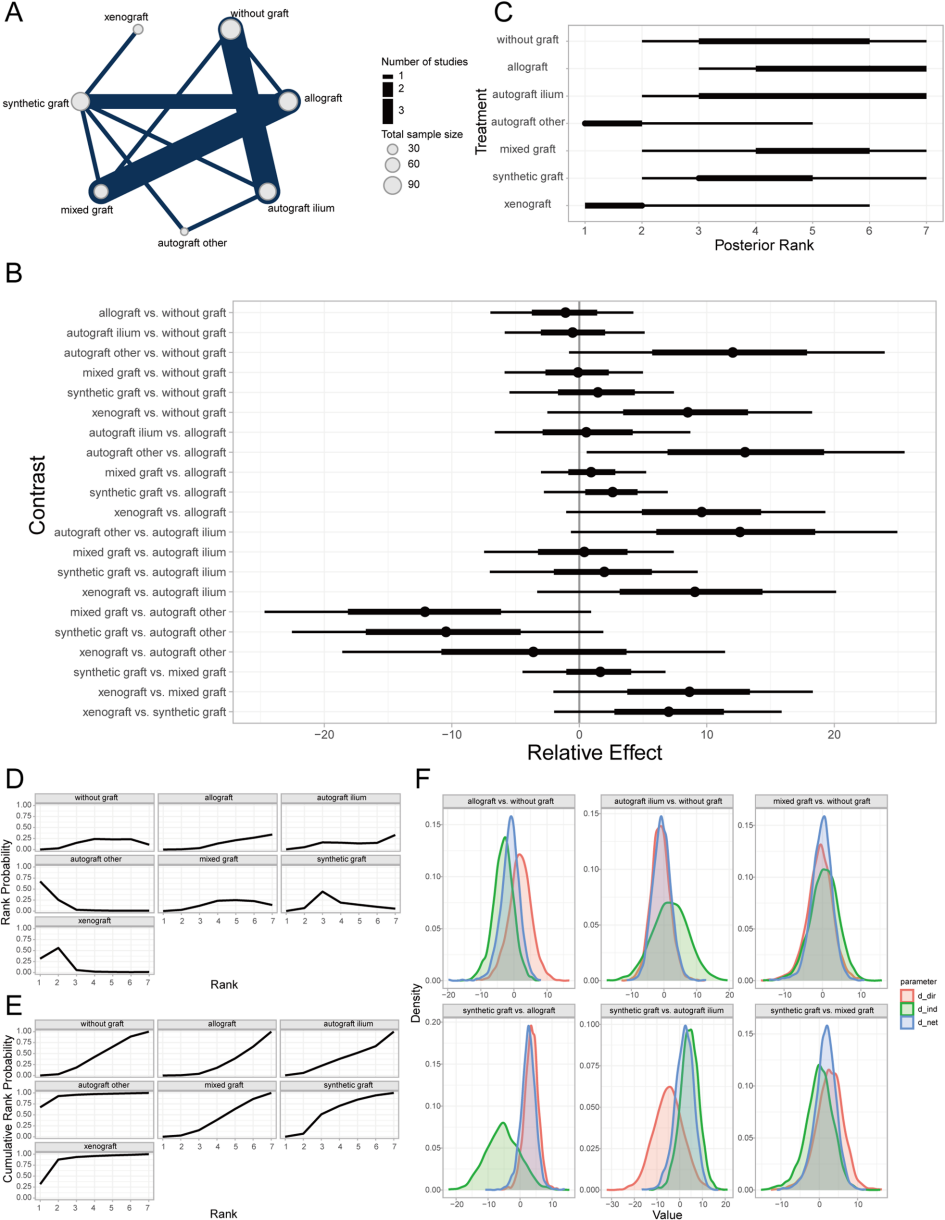
Prognostic analysis of different bone defect filling materials based on functional KSS. **A** Network evidence plot; **B** Forest plot of pairwise intervention comparisons; **C** Intervention ranking plot; **D** Probability of order plot for individual interventions; **E** Cumulative probability of order plot for individual interventions; **F** Local inconsistency analysis plot

The Functional Knee Society Score (KSS), a component of the broader KSS system, evaluates functional outcomes after High Tibial Osteotomy (HTO) [46]. Bayesian random-effects network meta-analysis showed that alternative autografts (e.g., autogenous tibial cortex) and xenografts were associated with higher Functional KSS scores compared to the non-graft group (Fig. 9A, B). The intervention rankings and probability of order analysis revealed that patients with alternative autografts had progressively better prognoses than those with xenogeneic, synthetic material grafts, non-graft, composite grafts, autogenous iliac bone grafts, and allografts (Fig. 9C–E). The local inconsistency analysis showed substantial overlap in the CrIs of direct and indirect comparisons, with all *P*-values exceeding 0.05 (Fig. 9F), indicating no significant local inconsistency in the reported findings.

### Comparison of complication outcomes among different bone defect fillers

#### Comparison of lateral cortical fracture outcomes across bone defect fillers

**Fig. 10:**
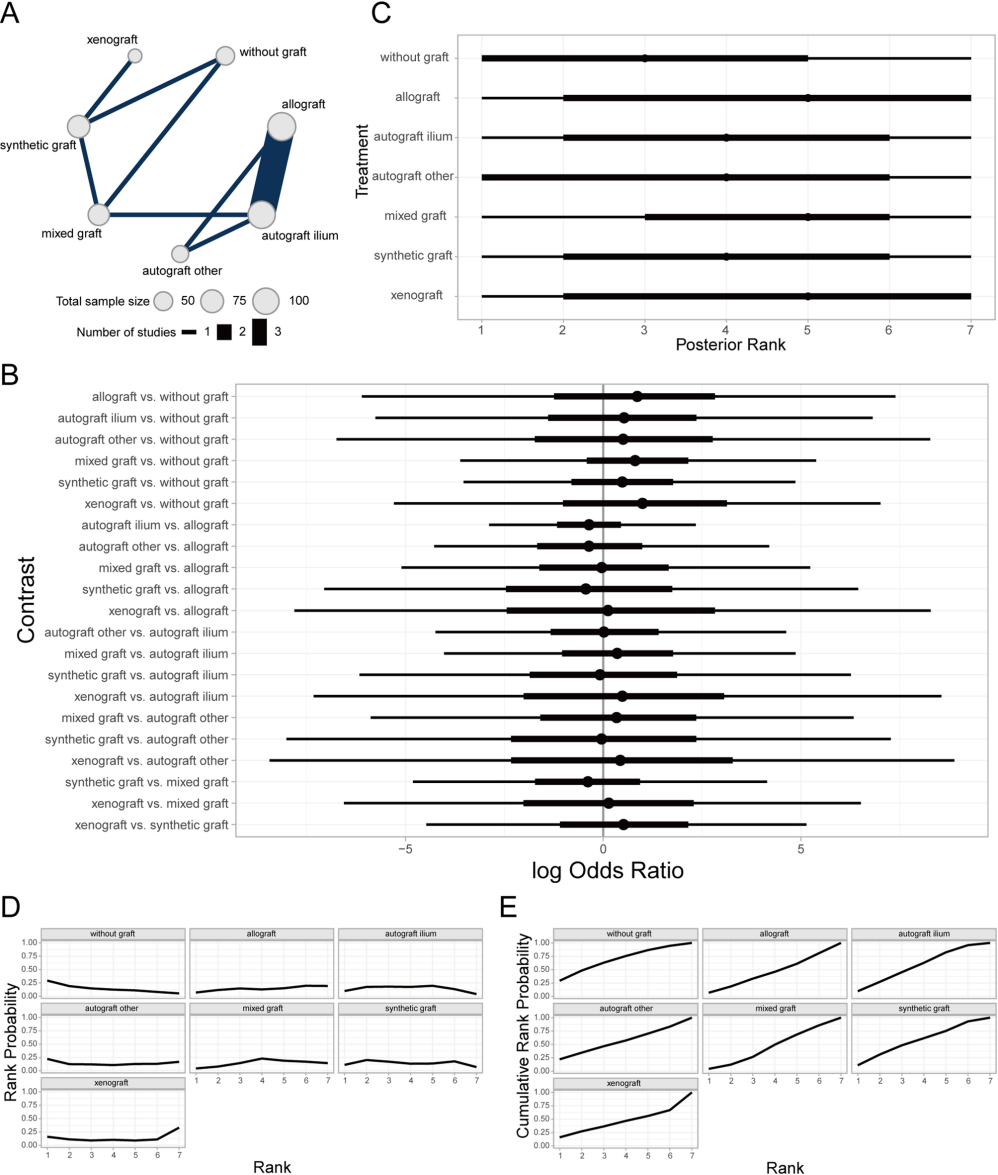
Comparison of lateral cortical fracture outcomes across different bone defect fillers. **A** Network of evidence; **B** Forest plot of pairwise comparisons; **C** Rankogram of interventions; **D** Surface under the cumulative ranking curve (SUCRA) plot; **E** Cumulative probability of ranking

Lateral cortical fracture, defined as a fracture of in the lateral cortex of the tibia post-HTO (High Tibial Osteotomy), can be a direct consequence of surgical manipulation or arise from altered bone stress during healing or improper patient activity [47]. This complication may impede postoperative recovery, prolong rehabilitation time, intensify postoperative pain, and potentially necessitate revision surgery or compromise surgical outcomes [48]. A Bayesian random-effects network meta-analysis revealed that, compared to the non-graft group, the mixed-graft group had a relatively higher incidence of lateral cortical fracture, albeit not statistically significant (Fig. 10A, B). Furthermore, ranking and order probability results suggested that non-graft patients had better outcomes than those receiving synthetic grafts, autogenous iliac bone grafts, autogenous grafts from other sites (e.g., tibial cortex), allografts, mixed grafts, and xenografts, in descending order (Fig. 10C–E).

#### Comparison of donor site morbidity outcomes among bone defect fillers

**Fig. 11:**
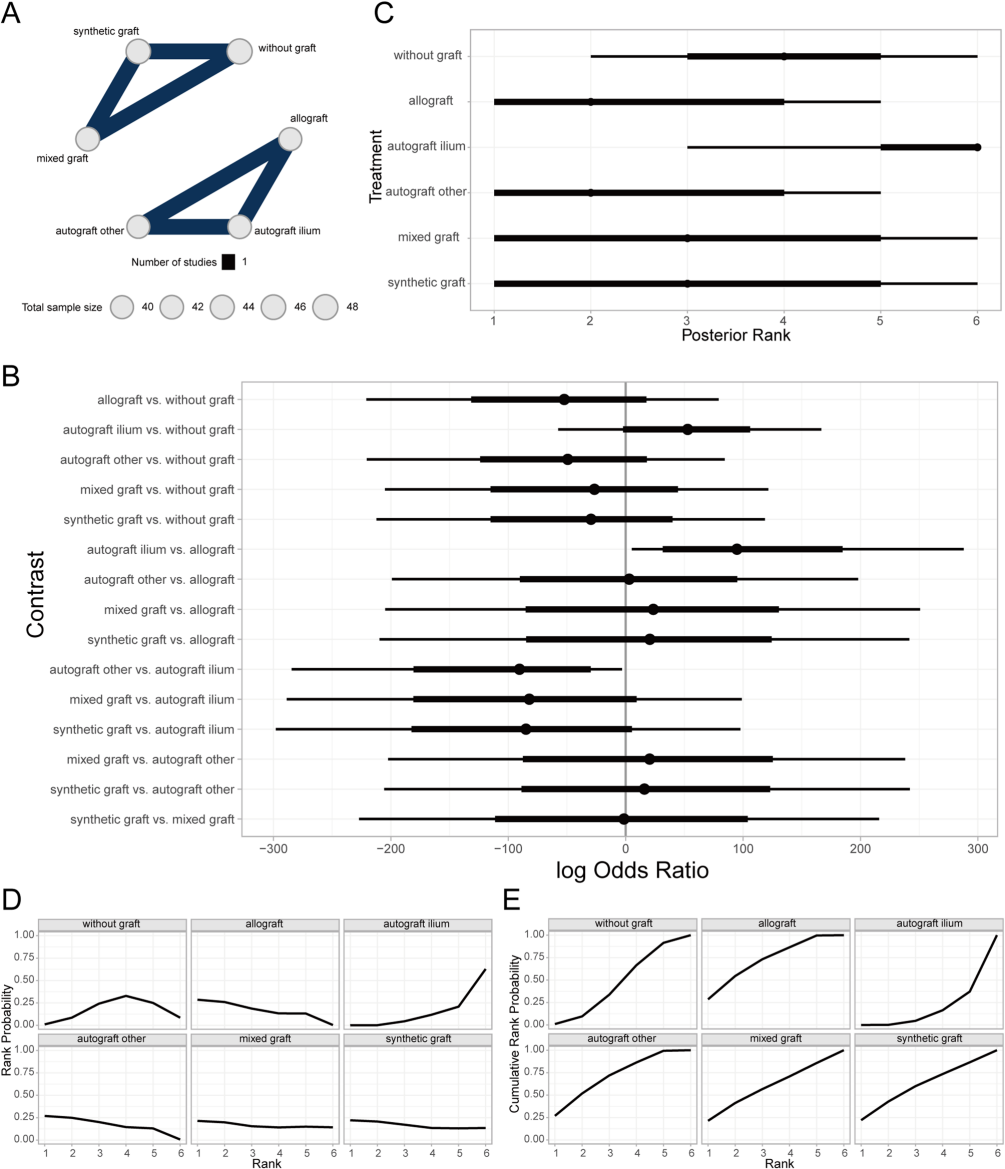
Comparison of donor site morbidity outcomes across different bone defect fillers. **A** Network of evidence; **B** Forest plot of pairwise comparisons; **C** Rankogram of interventions; **D** SUCRA plot; **E** Cumulative probability of ranking

Donor site morbidity in the context of HTO encompasses complications resulting from harvesting bone tissue or materials from other anatomical sites for grafting [49]. The Bayesian random-effects network meta-analysis demonstrated that autogenous iliac bone grafts were associated with a relatively higher donor site morbidity compared to the non-graft group (Fig. 11A, B). The rankings and probability of order results further suggested that allograft patients had better outcomes than those with autogenous grafts from other sites, synthetic grafts, mixed grafts, non-graft, and autogenous iliac bone grafts, in descending order (Fig. 11C–E).

#### Comparison of infection outcomes among bone defect fillers

**Fig. 12:**
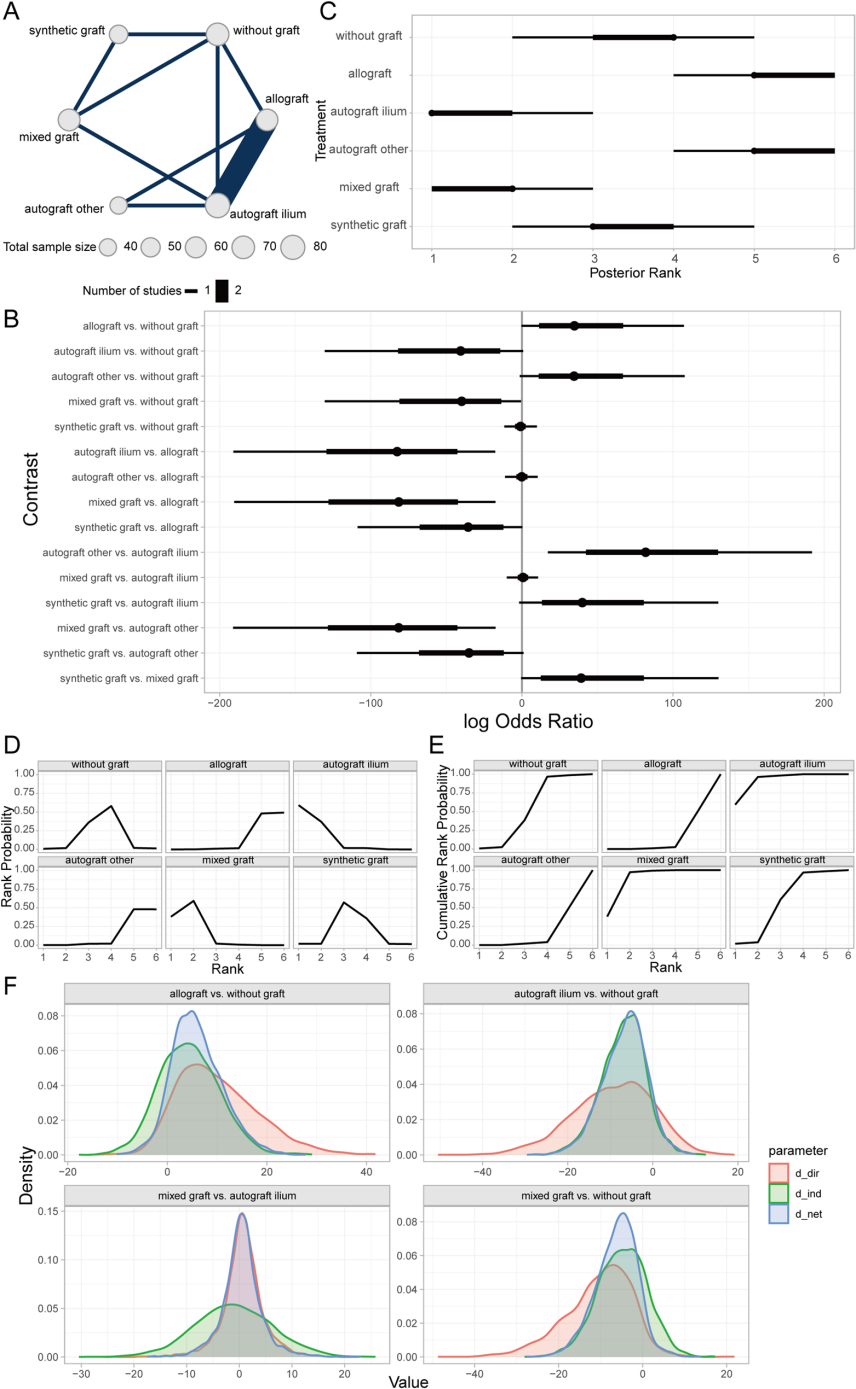
Comparison of infection outcomes across different bone defect fillers. **A** Network of evidence; **B** Forest plot of pairwise comparisons; **C** Rankogram of interventions; **D** SUCRA plot; **E** Cumulative probability of ranking; **F** Local inconsistency analysis plot

Infection after HTO denotes infectious lesions at the surgical site or elsewhere, typically caused by microorganisms such as bacteria or viruses, which may trigger local or systemic inflammation [50]. The Bayesian random-effects network meta-analysis found that autogenous iliac bone grafts and mixed grafts had relatively lower infection rates compared to the non-graft group, whereas autogenous grafts from other sites and allografts exhibited higher infection rates (Fig. 12A, B). The rankings and probability of order analysis indicated that autogenous iliac bone graft patients had better outcomes than those with mixed grafts, synthetic grafts, non-graft, allografts, and autogenous grafts from other sites (Fig. 12C–E). Additionally, local inconsistency analysis revealed that most CrIs for direct and indirect comparisons overlapped, with *P*-values > 0.05 (Fig. 12F), indicating no significant local inconsistency in the results.

#### Comparison of delayed union outcomes among different bone defect filling materials

**Fig. 13:**
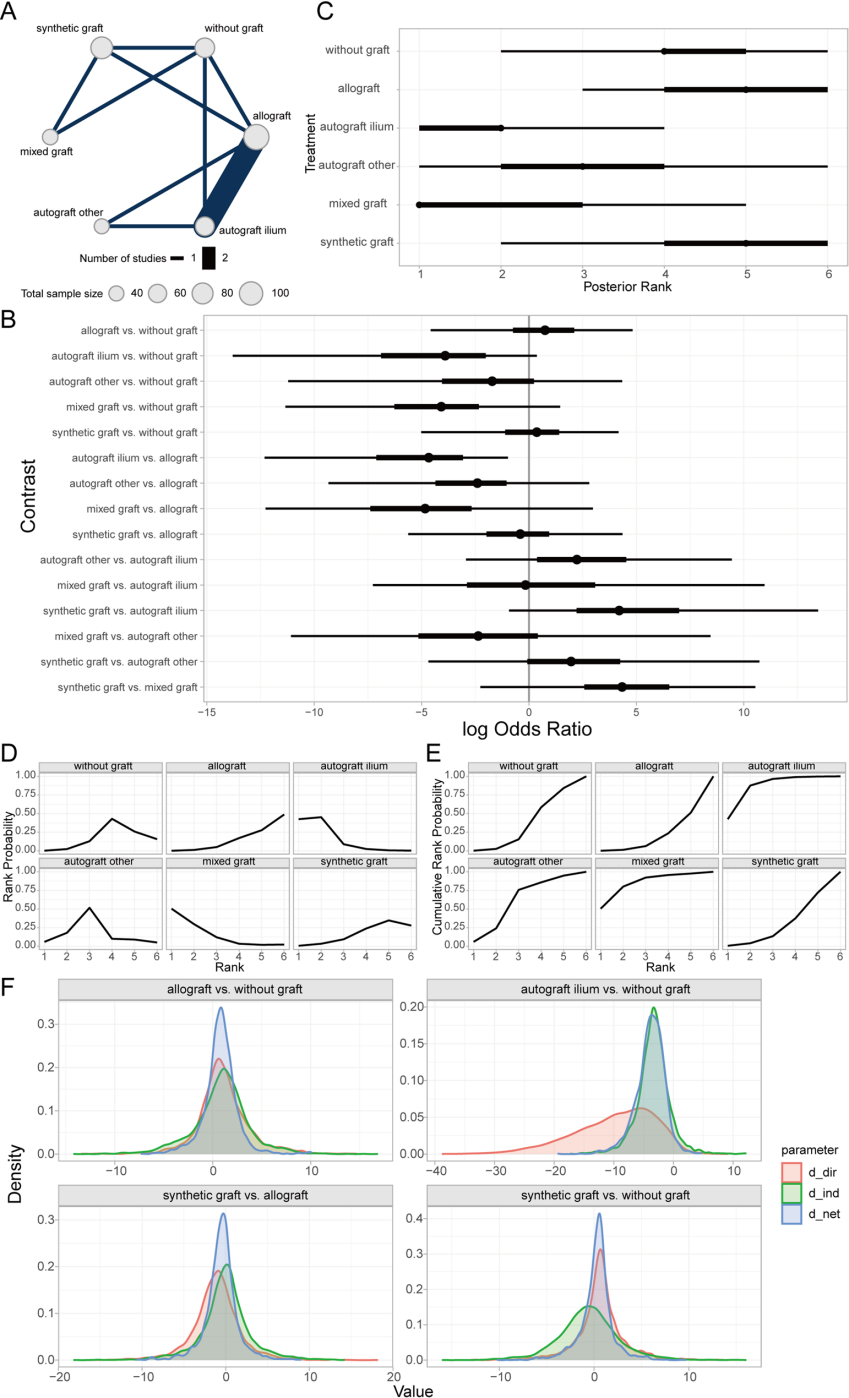
Comparison of delayed union outcomes among different bone defect filling materials. **A** Network evidence graph; **B** Forest plot for pairwise comparisons among interventions; **C** Intervention ranking plot; **D** Probability of order plot for each intervention; **E** Cumulative probability of order plot for each intervention; **F** Local inconsistency analysis results plot

Delayed union is characterized by fracture healing that is significantly prolonged beyond the normal expected timeframe, resulting in failure to achieve bony union at the fracture ends as anticipated. In the context of high tibial osteotomy (HTO), this typically presents as slow healing at the osteotomy site, requiring an extended period to achieve a stable bony union [51]. Our Bayesian random-effects network meta-analysis revealed that, compared to the non-graft group, both autogenous iliac bone grafting and combined grafting exhibited relatively lower rates of Delayed union (Fig. 13A, B). Furthermore, the ranking and probability of order analysis for each intervention indicated that the prognosis of patients undergoing combined grafting was sequentially superior to that of autogenous iliac bone grafting, autogenous alternative grafting (e.g., autogenous tibial cortex), no grafting, synthetic material grafting, and allogeneic bone grafting (Fig. 13C–E). Additionally, the results of the local inconsistency analysis suggested that the majority of the credible intervals (CrIs) for direct and indirect comparisons overlapped, with all *P*-values exceeding 0.05 (Fig. 13F). These findings collectively indicate the absence of significant local inconsistency in the aforementioned outcomes.

## Discussion

Guided by the enhanced recovery after surgery (ERAS) protocol, this study conducted a systematic evaluation of bone defect fillers in medial open-wedge high tibial osteotomy (MOWHTO)—a procedure increasingly performed within fast-track orthopedic rehabilitation [5]. ERAS emphasizes multimodal perioperative management (e.g., opioid-sparing analgesia, early mobilization) to reduce surgical stress and enhance recovery [52]. Within this paradigm, bone defect fillers act as biological scaffolds that critically influence osteointegration kinetics and weight-bearing timelines. Our analysis positions filler selection as a modifiable factor in postoperative rehabilitation trajectories. For instance, delayed bone union may necessitate prolonged restricted weight-bearing, directly conflicting with ERAS goals of early ambulation. This underscores a previously overlooked connection between biomaterial science and rehabilitation strategies.

Using a Bayesian network meta-analysis (NMA), this study demonstrated that bone defect filler selection significantly influences knee function recovery (e.g., Knee Society Score, KSS) and bone healing rates within one year, thereby modulating patient recovery trajectories. This observation aligns with ERAS principles [6]. Adhering to PRISMA-2020 NMA guidelines, we aimed to ensure methodological rigor [53, 54]. Comprehensive searches across multiple databases enabled a systematic evaluation of filler efficacy, offering insights for ERAS integration in MOWHTO. These findings may inform clinical practice, though the evidence requires cautious interpretation. It is noteworthy that for patients with medial compartment knee osteoarthritis (MCKOA) and severe concomitant cartilage injuries, recent studies have demonstrated that combining limb realignment with cartilage repair techniques—such as autologous chondrocyte implantation—can simultaneously address abnormal mechanical loading and cartilage defects, thereby promoting more comprehensive recovery of joint function [14]. This integrated approach offers novel perspectives for the management of complex knee disorders and underscores the need for future research to focus on the synergistic effects of multimodal combined treatment strategies [16].

The primary novelty of this study lies in its comparative analysis of multiple bone defect fillers for MOWHTO outcomes. By systematically assessing autogenous iliac grafts, synthetics, allografts, and composites, we addressed a gap in previous research limited to single-filler comparisons. Our results demonstrated that autogenous iliac bone grafts appeared to show advantages in bone healing rates and Lysholm knee scores, consistent with ERAS goals and reported biomechanical properties [7]. However, their association with donor site morbidity warrants caution. Synthetic materials showed favorable trends in WOMAC scores, potentially aiding functional recovery [27, 32]. Combining direct and indirect evidence via Bayesian NMA provided a balanced view of filler trade-offs, supporting nuanced clinical decision-making.

This study has several limitations that affect the certainty of the evidence. First, the inclusion of both randomized trials (RCTs, n = 10) and non-randomized studies (NCTs, n = 19, total participants = 1549) introduced heterogeneity due to variations in patient demographics, filler types, follow-up durations, and outcome measures. This heterogeneity may introduce bias into pooled estimates despite Bayesian adjustments. Second, reliance on NMA is inherently constrained by the quality of included studies; inconsistencies in protocols (e.g., differing KSS/WOMAC thresholds) could impact reliability. Third, self-reported outcome measures (e.g., WOMAC, KSS) are susceptible to patient-reported bias. Fourth, the one-year follow-up may be insufficient to capture long-term outcomes. Future research should prioritize large, multicenter RCTs with longer follow-ups to validate findings and address limitations.

## Conclusion

Aligned with enhanced recovery after surgery (ERAS) principles, this study indicates that bone graft material selection in medial open-wedge high tibial osteotomy (MOWHTO) might influence surgical outcomes and recovery trajectories. Autogenous iliac bone grafting demonstrated benefits for bone healing and knee function but carried risks of donor site morbidity—highlighting a critical trade-off. Synthetic materials and allografts may represent viable alternatives with potential to support functional recovery, though evidence remains preliminary. Future research should focus on large-scale, multicenter RCTs to validate these observations and evaluate long-term efficacy through extended follow-ups. Such efforts would strengthen the evidence base for clinical decision-making in MOWHTO.

## Supplementary Information

Below is the link to the electronic supplementary material.

Supplementary Material 1.

Supplementary Material 2.

Supplementary Material 3.

Supplementary Material 4.

## Data Availability

No datasets were generated or analysed during the current study.

## Abbreviations

HTO: High tibial osteotomy
MCKOA: Medial compartment knee osteoarthritis
ERAS: Enhanced recovery after surgery
RCTs: Randomized controlled trials
NCTs: Non-randomized comparative trials
KOA: Knee osteoarthritis
MOWHTO: Medial opening-wedge high tibial osteotomy
NMA: Network meta-analysis
PRISMA: Preferred reporting items for systematic reviews and meta-analyses
AMSTAR: Assessing the methodological quality of systematic reviews
TBU: Time to bone union
PCBU: Percentage of complete bone union
LOC: Loss of correction
LKS: Lysholm knee score
WOMAC: Western ontario and mcmasters universities score
OKSS: Objective knee society scoring
FKSS: Functional knee society scoring
LCF: Lateral cortical fracture
DSM: Donor site morbidity
DU: Delayed union
BMI: Body mass index
CrIs: Credible intervals
Ors: Odds ratios
