# Supplementary material for: Network meta-analysis of high tibial osteotomy combined with different bone defect filler materials for medial compartment knee osteoarthritis
Source: J Orthop Surg Res. 2025 Oct 21;20:901. doi: 10.1186/s13018-025-06306-w (PMC12538742; doi:10.1186/s13018-025-06306-w)
Supplement: Supplementary file 1 — Supplementary Material 1. [file 13018_2025_6306_MOESM1_ESM.docx]

**Supplementary materials**


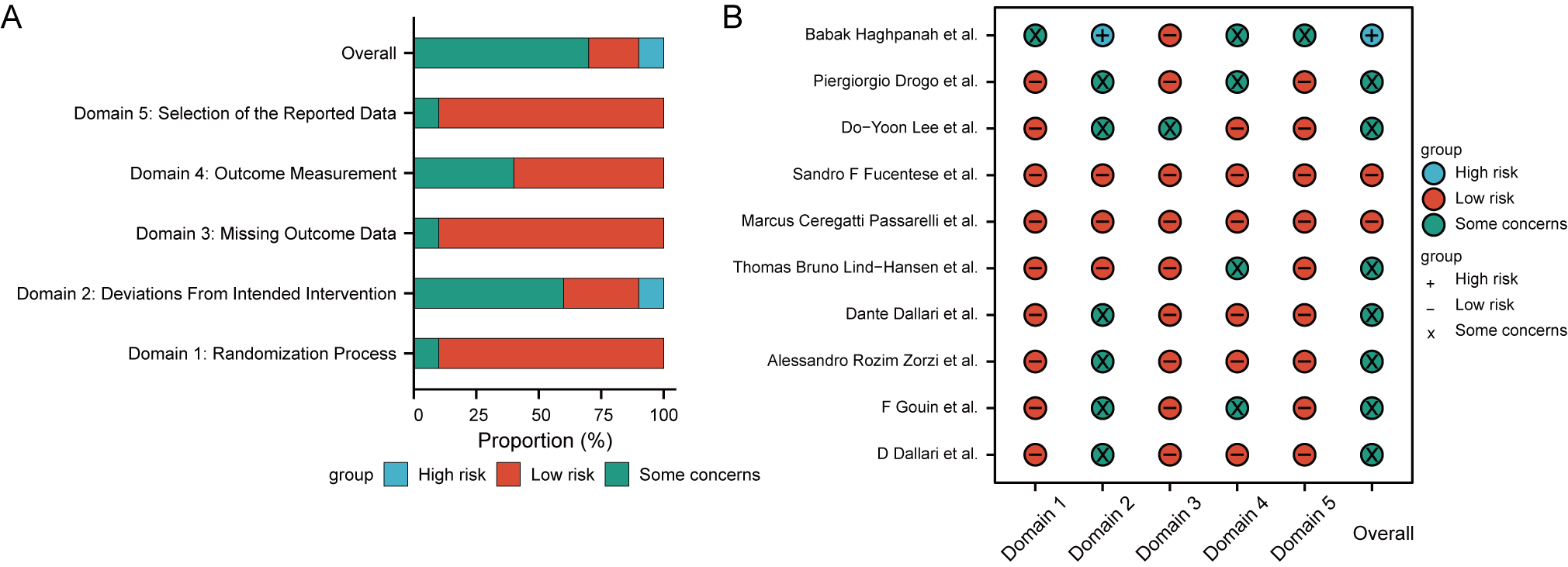


Supplementary Figure 1. Quality and Risk of Bias Assessment of Included Studies. (A) Summary plot generated using the Cochrane Risk of Bias tool for Randomized Controlled Trials (RoB 2.0); (B) Traffic-light plot generated using the Cochrane Risk of Bias tool for Randomized Controlled Trials (RoB 2.0).


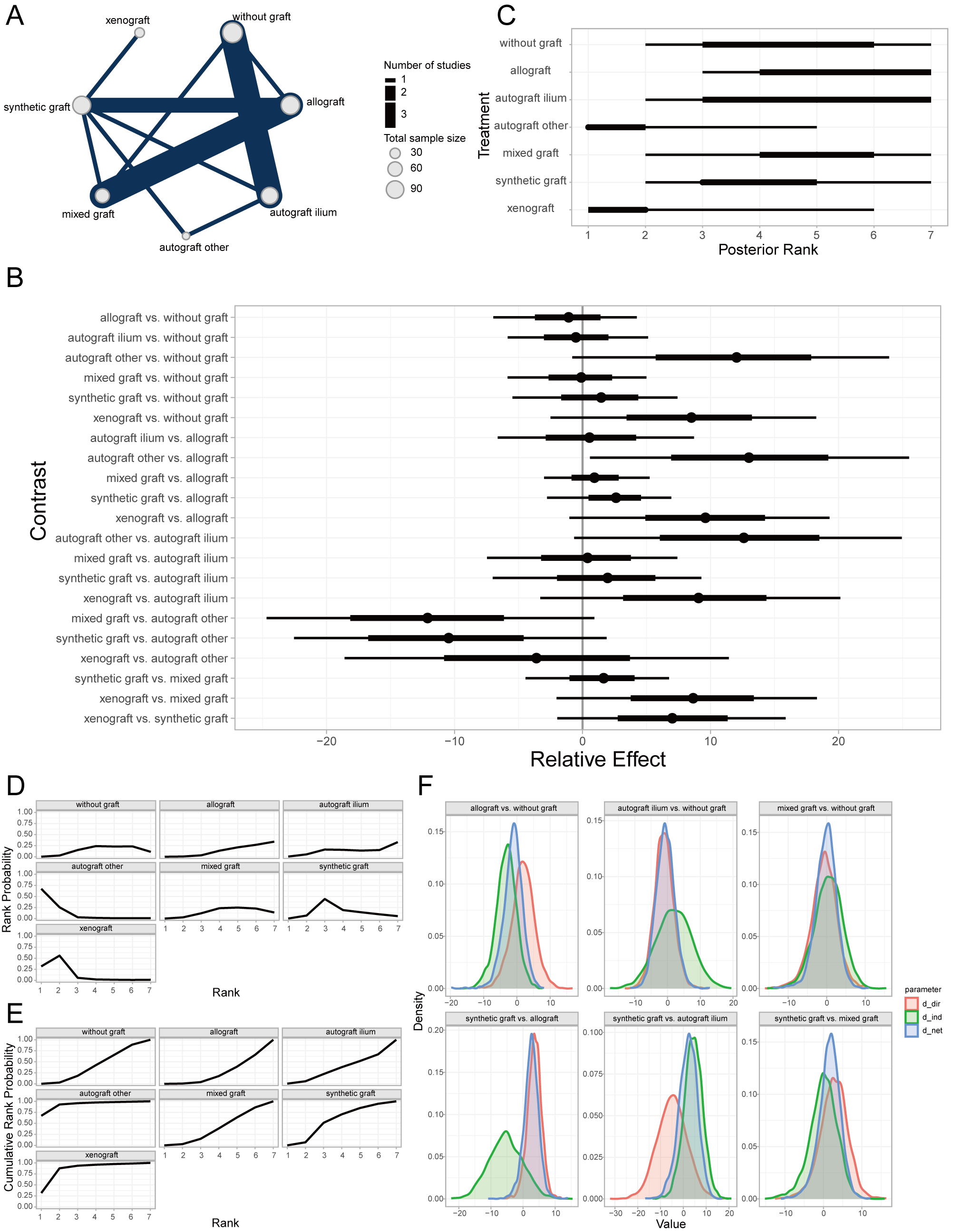


Supplementary figure 2. Prognostic Analysis of Different Bone Defect Filling Materials Based on Functional KSS. (A) Network evidence plot; (B) Forest plot of pairwise intervention comparisons; (C) Intervention ranking plot; (D) Probability of order plot for individual interventions; (E) Cumulative probability of order plot for individual interventions; (F) Local inconsistency analysis plot.


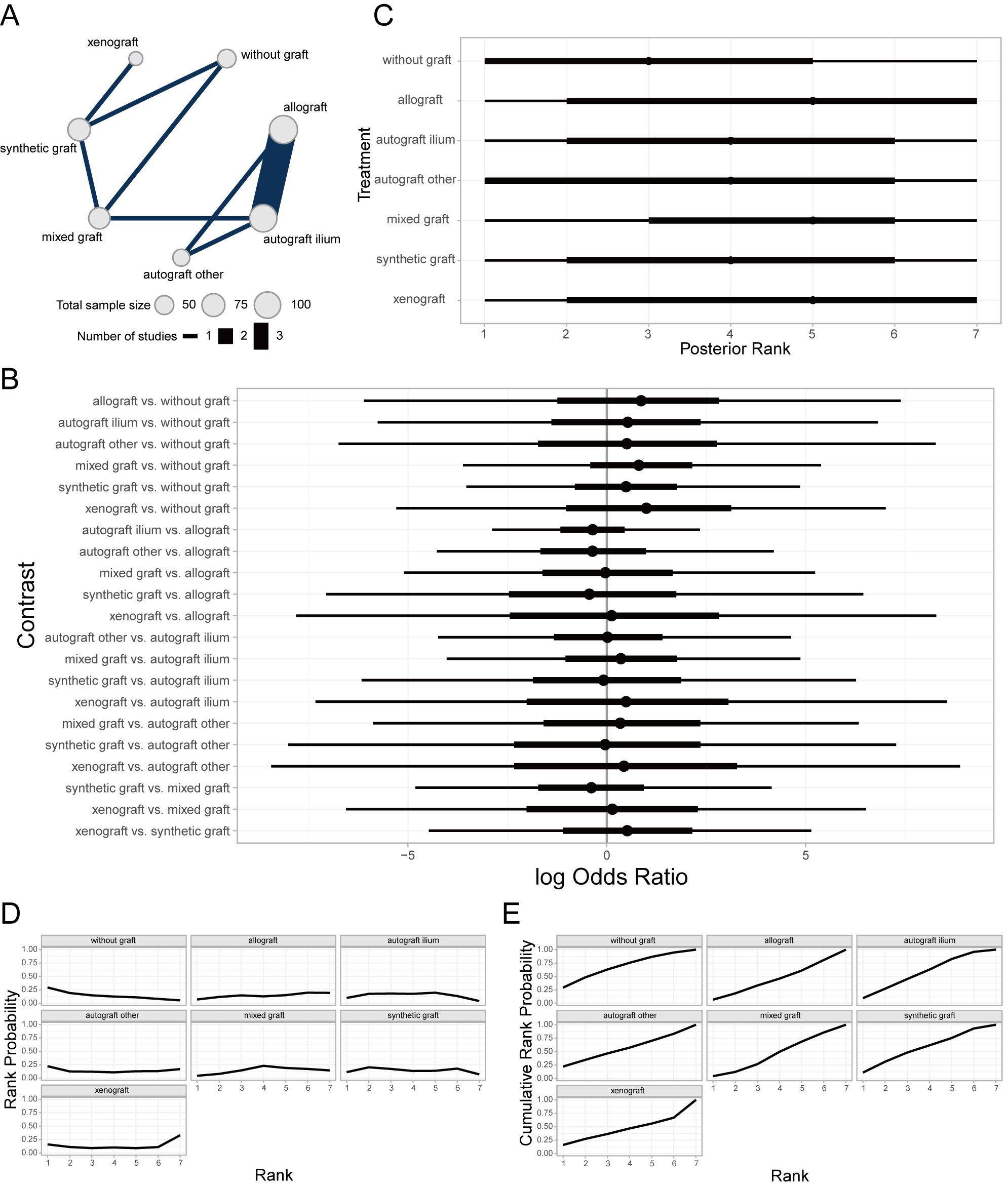


Supplementary figure 3. Comparison of Lateral Cortical Fracture Outcomes Across Different Bone Defect Fillers. (A) Network of evidence; (B) Forest plot of pairwise comparisons; (C) Rankogram of interventions; (D) Surface under the cumulative ranking curve (SUCRA) plot; (E) Cumulative probability of ranking.


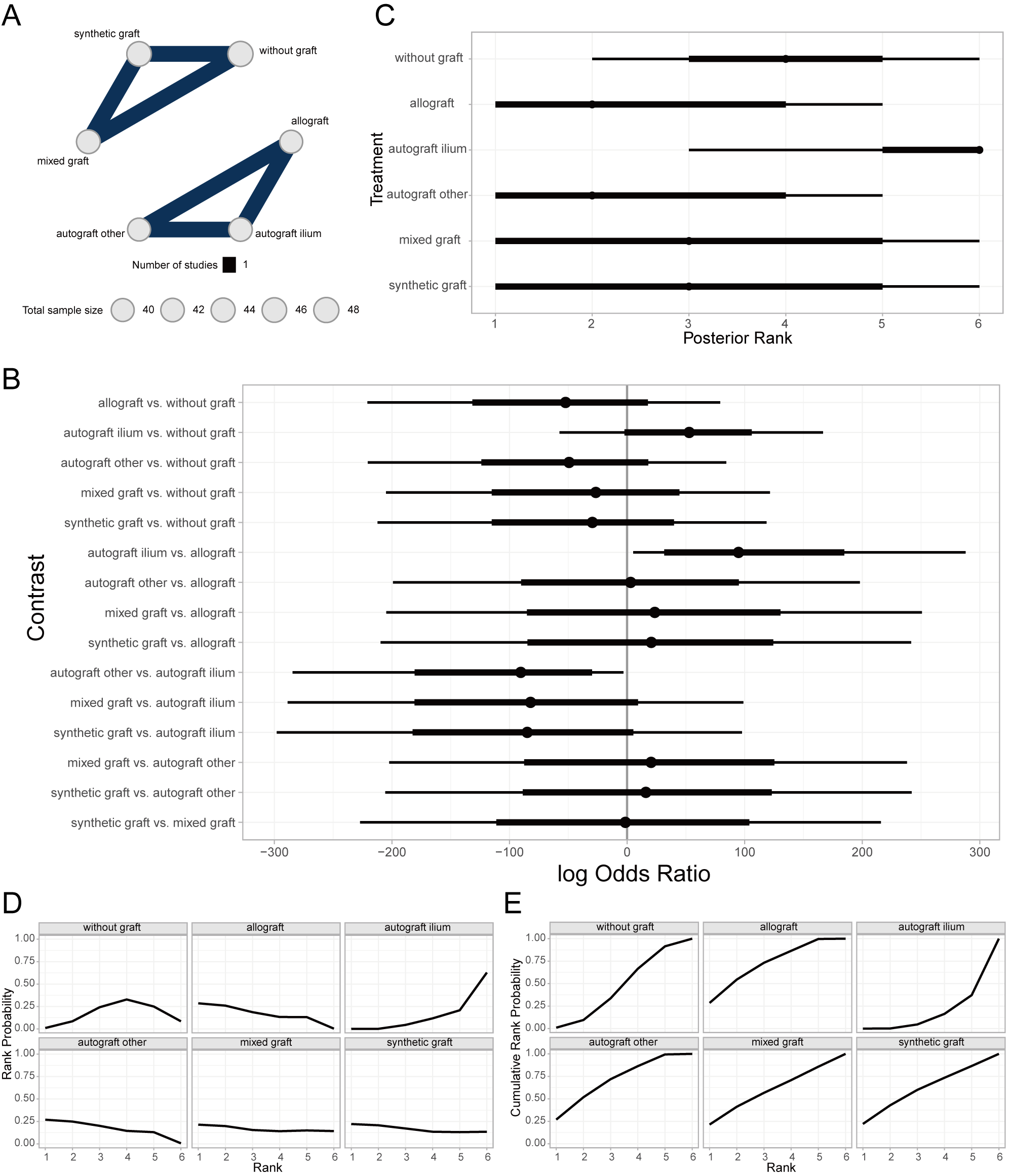


Supplementary figure 4. Comparison of Donor Site Morbidity Outcomes Across Different Bone Defect Fillers. (A) Network of evidence; (B) Forest plot of pairwise comparisons; (C) Rankogram of interventions; (D) SUCRA plot; (E) Cumulative probability of ranking.


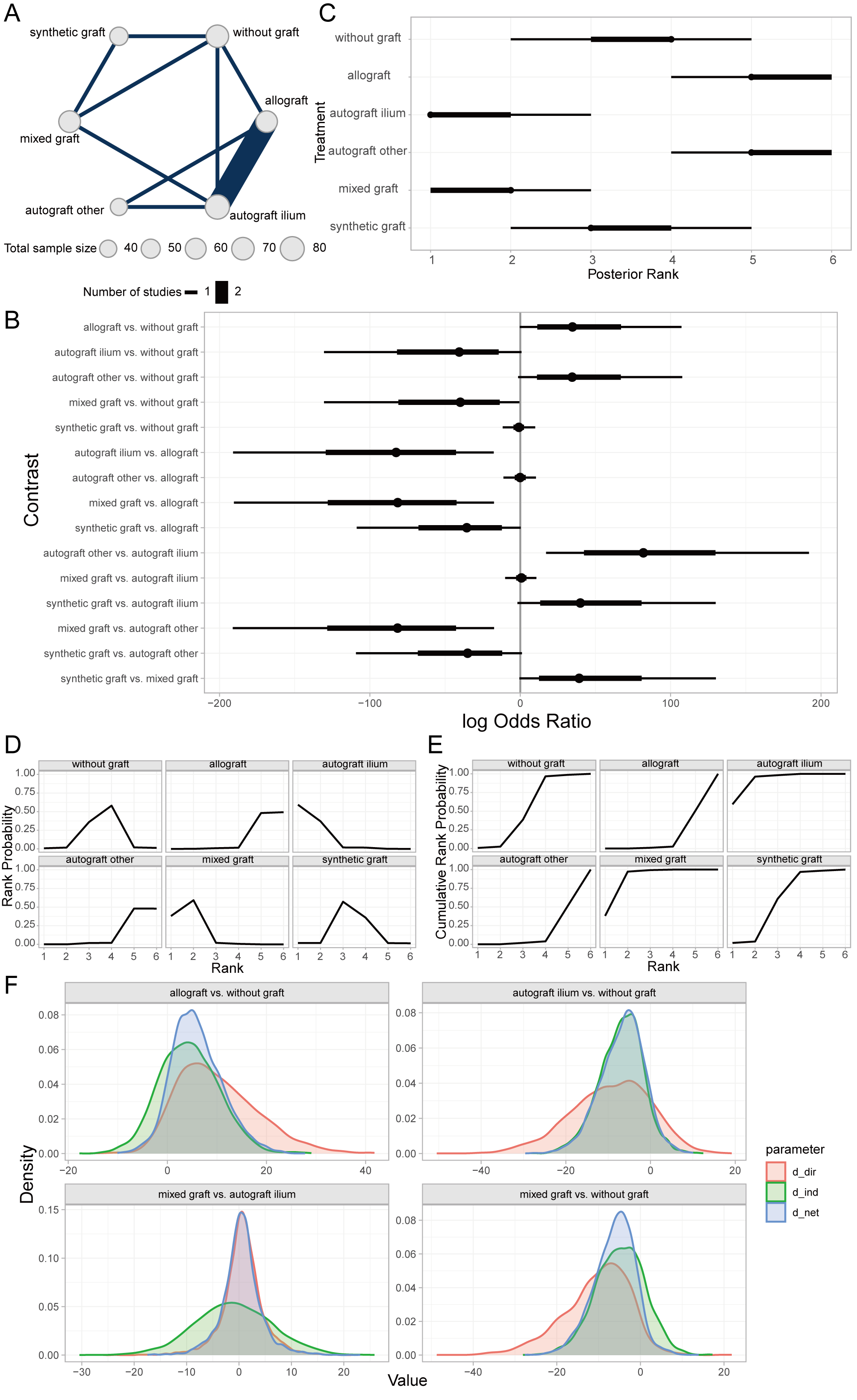


Supplementary figure 5. Comparison of Infection Outcomes Across Different Bone Defect Fillers. (A) Network of evidence; (B) Forest plot of pairwise comparisons; (C) Rankogram of interventions; (D) SUCRA plot; (E) Cumulative probability of ranking; (F) Local inconsistency analysis plot.


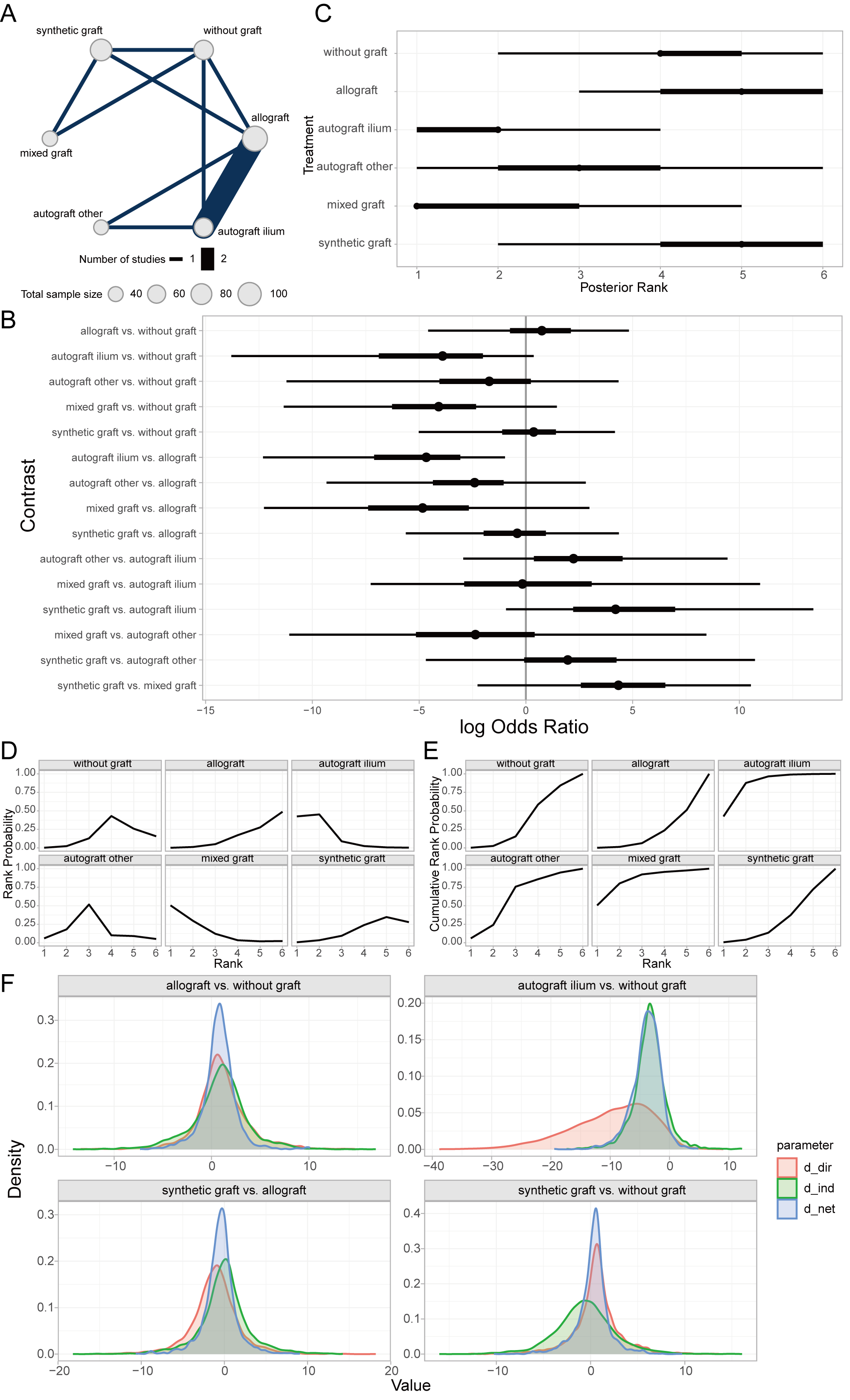


Supplementary figure 6. Comparison of Delayed Union Outcomes among Different Bone Defect Filling Materials. (A) Network evidence graph; (B) Forest plot for pairwise comparisons among interventions; (C) Intervention ranking plot; (D) Probability of order plot for each intervention; (E) Cumulative probability of order plot for each intervention; (F) Local inconsistency analysis results plot.
